# Abnormalities of Thyroid Hormone Metabolism during Systemic Illness: The Low T3 Syndrome in Different Clinical Settings

**DOI:** 10.1155/2016/2157583

**Published:** 2016-10-10

**Authors:** Arnaldo Moura Neto, Denise Engelbrecht Zantut-Wittmann

**Affiliations:** Division of Endocrinology, Department of Clinical Medicine, Faculty of Medical Sciences, University of Campinas, Campinas, SP, Brazil

## Abstract

Thyroid hormone abnormalities are common in critically ill patients. For over three decades, a mild form of these abnormalities has been described in patients with several diseases under outpatient care. These alterations in thyroid hormone economy are a part of the nonthyroidal illness and keep an important relationship with prognosis in most cases. The main feature of this syndrome is a fall in free triiodothyronine (T3) levels with normal thyrotropin (TSH). Free thyroxin (T4) and reverse T3 levels vary according to the underlying disease. The importance of recognizing this condition in such patients is evident to physicians practicing in a variety of specialties, especially general medicine, to avoid misdiagnosing the much more common primary thyroid dysfunctions and indicating treatments that are often not beneficial. This review focuses on the most common chronic diseases already known to present with alterations in serum thyroid hormone levels. A short review of the common pathophysiology of the nonthyroidal illness is followed by the clinical and laboratorial presentation in each condition. Finally, a clinical case vignette and a brief summary on the evidence about treatment of the nonthyroidal illness and on the future research topics to be addressed are presented.

## 1. Introduction

The low T3 (triiodothyronine) syndrome, also known as the euthyroid sick syndrome or the nonthyroidal illness syndrome (NTIS), was initially described in the 1970s. It represents a state of alterations in thyroid hormone (TH) economy classically present in critically ill patients, particularly those admitted to intensive care units [1]. These abnormalities are, by definition, not related to intrinsic diseases of the hypothalamus-pituitary-thyroid axis but rather represent imbalances in thyroid hormone production, metabolism, and action [2].

The hallmark of this syndrome is a fall in serum T3 levels that may be accompanied by a drop in serum thyroxine (T4) levels. Serum thyrotropin (TSH) is usually normal but may be slightly increased or even decreased. In the recent decades, the syndrome has also been described in patients with chronic conditions and under outpatient care [3–8]. The importance of recognizing this condition in such patients is evident to physicians practicing in a variety of specialties, especially general medicine, in order to avoid misdiagnosing the much more common primary thyroid dysfunctions and indicating treatments that are often not beneficial.

## 2. Laboratorial Presentation

Much information about the pathophysiology and the main laboratorial abnormalities of NTIS is derived from animal models or patients admitted to the intensive care unit. In such patients, TH abnormalities usually show two distinct temporal phases. In the first phase, acute modifications in peripheral thyroid hormone metabolism predominate. In the second phase, disturbances of neuroendocrine origin predominate [9].

In patients under ambulatory care, the presentation often carries components of both phases. The fall in T3 levels is always present and the diagnosis should be suspected if low T3 presents concurrently with low or normal TSH. There is often a rise in reverse T3 (rT3) levels as well, and the decrease in T3/rT3 relationship is considered the most sensitive parameter for diagnosis of NTIS [10]. This is somewhat complicated in routine clinical care because rT3 is not part of thyroid hormone profiles. The value of free T4 measurements in NTIS is a matter of debate; as results are strongly influenced by the laboratorial method employed [11]. The association between TH and prognosis is conserved among various noncritical conditions [4, 12, 13]. The main laboratorial abnormalities that may be identified in the most common clinical situations associated with NTIS will be discussed in the appropriate sections below.

## 3. Pathophysiology

In patients with noncritical diseases peripheral abnormalities in hormone conversion predominate. The abnormalities are better reflected by the relations T3/rT3 and FT3/rT3, corroborating the action of peripheral mechanisms favouring decreased thyroid hormone activation and increased inactivation [14, 15].

The peripheral metabolism of TH is determined by the action of the three selenodeiodinases (D1, D2, and D3) that catalyse the interconversion of different iodothyronines. Studies in critically ill patients have shown decreased activity of D1 in liver and skeletal muscle [14], increased D2 activity in skeletal muscle [16], and increased activity of D3 in patients with acute myocardial infarction [15]. The diminished production of T3 from T4 resultant of low D1 activity combined with increased rT3 production from increased D3 activity generates the classical pattern of low T3 and increased rT3, while also explaining why in some conditions higher T4 levels may be found [17].

Additionally, abnormal production of thyroid binding globulin (TBG) is a potential cause for thyroid hormone alterations in patients with NTIS, especially if total T3 or T4 is being measured. Usually, patients with NTIS have low TBG levels [18]. In some cases, such as nephrotic syndrome, massive protein loss can be a contributor for this [8]. Diseases that affect the liver and patients with HIV may show elevated TBG levels that would make the interpretation of laboratorial data more difficult [19, 20]. The advent of free T3 measurement almost eliminated this trouble since even conditions with high TBG levels show low serum free T3 during NTIS [21]. Patients treated with proinflammatory cytokines also show decreased TBG levels, which are normalized after drug interruption [22].

Proinflammatory cytokines are often elevated in NTIS and have been demonstrated to correlate inversely with thyroid hormone levels in the critically ill [23, 24] as well as in patients with chronic diseases [4, 17, 25].

Furthermore, these cytokines are possibly implicated in the suppression of hypothalamic-pituitary axis often seen in NTIS [25]. Production of thyrotropin release hormone (TRH) mRNA is decreased in patients with NTIS but not in those who died of immediate external causes [26]. Increased pituitary activity of D2 has been demonstrated [16] and may be a contributor for this abnormality [27]. More T3 produced locally by this enzyme could render the pituitary euthyroid even in face of a generalized hypothyroid state, with low circulating levels of T3 [27–29]. Since most chronic ambulatory diseases carry a strong inflammatory component, it is very likely that many (if not all) of these mechanisms are present in these situations. The relationship between proinflammatory cytokines and thyroid hormone levels has been shown in patients with chronic obstructive pulmonary diseases and diabetes mellitus [4, 17, 30].

An important factor that is worth mentioning is that patients with chronic systemic diseases are often under treatment with several drugs that can affect thyroid hormone metabolism [31]. Among examples of situations where systemic diseases that affect TH metabolism coexist with the use of medications that also alter TH metabolism are patients with heart or liver diseases taking beta-blockers [32, 33], those with heart failure receiving amiodarone [34], and patients with psychiatric conditions under treatment with lithium and/or drugs that affect the hepatic metabolism of TH [31, 35]. A discussion on this topic is beyond the scope of this review, but this caveat should nonetheless be taken into account when interpreting thyroid function tests in patients with chronic conditions.

## 4. Nonthyroidal Illness in Different Clinical Settings

NTIS has been reported in a variety of situations, even when patients are well enough to be seen in an outpatient setting. In this section, we review the most common conditions associated with abnormalities in thyroid hormone levels that are compatible with a mild or atypical form of NTIS.  Table 1 summarizes the laboratorial abnormalities found in these situations.

### 4.1. Caloric Deprivation

Changes in TH levels during prolonged fasting are linked to two main factors: changes in basal energy expenditure and leptin levels.

During caloric deprivation, the fall in serum T3 is believed to be an adaptative response directed to saving energy and protein for enduring an acute stress stimulus [36]. It results from peripheral inhibition of T4 metabolism and decreased TSH response to hypothalamic TRH. It has been shown that in a hypocaloric diet a fall in T3 levels occurs, with a simultaneous transient increase in free T4 [37]. Increased rT3 is observed in the first two weeks, followed by normalization thereafter [38]. Normalization of rT3 levels occurred in parallel to decreased T3 concentrations [39, 40]. The elevation in serum rT3 levels is related to decreased catabolism by deiodinases and not increased production from T4 [37]. The fall in T3 is a result of decreased conversion from T4. Decreased ATP availability during fasting could impair T4 uptake by the liver as well as peripheral deiodination. Total and free T4 are within normal concentrations [41].

More recent evidence shows that, in addition to a decreased conversion of T4 to T3 during fasting, suppression of the hypothalamic-pituitary-thyroid axis is seen [40, 42, 43]. Leptin is an important factor in this regard, because its levels fall in concert with weight loss [44–46]. Leptin was shown to stimulate TSH secretion, and this finding may help to explain the increased TSH levels often found in obese individuals [45, 46]. Patients who have a defective leptin receptor due to genetic mutations show reduced pituitary hormone secretion, with delayed puberty and diminished TSH secretion [47]. Prevention of the starvation-mediated fall in leptin levels by administration of exogenous leptin can significantly blunt the abnormalities found in TH levels in this situation [45]. It appears that in humans, as opposed to what is seen in animal models, a minimal serum level of leptin is necessary for adequate pituitary function and maintenance of leptin above this threshold prevents the fall in thyroid hormone levels as well as other hormonal axes commonly seen during prolonged fasting [46]. On the other hand, some recent animal models of NTIS have shown that intrahepatic D3 activity is increased independently of autonomic nerve function [48].

### 4.2. HIV Infection

HIV infection and NTIS are related not only by the chronic infection status, but also by the catabolic state resulting from the disease itself and its opportunistic infections [49–51]. A fall in serum T3 levels is found in up to 20% of patients carrying the virus and 50% of those harbouring an opportunistic infection [20, 50, 52]. Some particularities are distinctive of this group of patients. Lower T3 levels concomitant with high TBG levels are often seen in this population [53]. Additionally, TBG levels increase as the disease progresses, but patients with poor prognosis usually have unchanged levels [54]. Another interesting finding, characteristic of this population, is low rT3 levels [55]. The low rT3 usually rises to normal levels upon hospitalization due to opportunistic infections [52].

There are several pitfalls other than NTIS that can coexist in a patient with HIV infection when analysing the results of thyroid function tests, such as thyroid infiltration by opportunistic pathogens (e.g.,* P. jirovecii*), weight loss, medications, and immune reconstitution syndrome [20, 52]. The prevalence of antithyroid antibodies, although low, increases after treatment and the consequent immune reconstitution and may be a potential confounder [56]. Thyroid function abnormalities are more frequent in patients under highly active antiretroviral therapy (HAART). The most common abnormality is subclinical hypothyroidism and FT4 is lower when compared to control subjects. In one study, HAART and particularly the use of stavudine were associated with subclinical hypothyroidism [56].

Weight loss is common in HIV patients and one study found that the most malnourished patients presented the lowest serum T3 [51]. Patients are as a rule clinically euthyroid and abnormalities in thyroid hormone levels are probably a reflection of disease severity [57].

### 4.3. Heart Diseases

Thyroid hormones are important modulators of several cardiac functions such as heart rate, cardiac output, systemic vascular resistance, and inotropism [58]. Abnormalities in thyroid hormone levels are frequently seen in situations of cardiac ischemia and congestive heart failure and after bypass surgery [59–61].

In cases of acute myocardial infarction, a fall in T3, T4, and TSH levels and an increase in rT3 have been reported. The relation rT3/TT3 is proportional to the severity of the case [62]. The total and free forms of T3 are also low after cardiac arrest caused by ischemia when compared to patients with noncomplicated myocardial infarction. Furthermore, patients who experienced more prolonged cardiac arrest showed lower TT3 and FT3 levels than those with shorter resuscitation time [62]. Additionally, thyroid function tests normalize after two weeks in patients who fully recover [62]. Oxidative stress probably plays a major role in the pathophysiology of thyroid hormone abnormalities in acute myocardial ischemia, as a small clinical trial demonstrated the ability of an anti-inflammatory medication to prevent NTIS in this setting [63].

In congestive heart failure, the prevalence of NTIS is around 18% [60] but can be as high as 23% [64]. Patients with higher severity scores usually develop more pronounced abnormalities in thyroid function tests than those less symptomatic. Low T3 concentrations were associated with higher mortality rates in patients hospitalized for heart failure, and serum free T3 concentrations were stronger predictors of mortality than established risk factors such as LDL-cholesterol, age, and left ventricular ejection fraction. T3 levels correlated with the New York Heart Association classification system [12].

### 4.4. Kidney Diseases

The kidney has an important role in the metabolism and excretion of thyroid hormones. Therefore, it is not surprising that kidney diseases can cause abnormalities in thyroid hormone axis [65].

In nephrotic syndrome, when proteinuria is greater than 3 g/24 hours with concomitant hypoalbuminemia, hypercholesterolemia, and oedema, serum T3 concentrations are low. Urinary loss of TBG, among other proteins, could justify such alterations. However, on patients with nephrotic syndrome but preserved renal function, TBG concentrations are within normal limits, falling only when there is impaired renal function [8]. Reverse T3 is typically normal, contrasting with other situations of NTIS when rT3 is often elevated [8]. Free T3 and T4 are usually normal and thyroid hormone supplementation is reserved only for situations of increased TSH, as a consequence of excessive urinary loss of thyroid hormones, or if low T4 is present because of the use of high dose corticosteroids for treatment of nephrotic syndrome [8].

In cases of terminal kidney disease, the almost complete loss of renal filtration alters the hypothalamic-pituitary-thyroid axis and causes abnormalities in peripheral thyroid hormone metabolism [65]. Like other clinical situations where NTIS occur, a decrease in T4 conversion to T3 with resultant low serum T3 is seen [66]. Similarly to what is observed in congestive heart failure, lower serum T3 levels predict mortality in patients under haemodialysis [13]. Serum rT3 levels are often normal, as in cases of nephrotic syndrome, and conversion of T4 to rT3 is unchanged [8, 67]. Total and free T4 are usually within reference ranges or mildly decreased. Free T4 can be mildly elevated in situations of heparin use to avoid blood clotting in the haemodialysis machine [68]. Haemodialysis does not correct the thyroid hormone imbalances of kidney failure, but this can be achieved with renal transplantation [65, 69].

### 4.5. Liver Disease

Normal hepatic function is essential to adequate metabolism of thyroid hormones. The liver is the main organ responsible for conversion of T4 to T3 (by the action of type 1 deiodinase), synthesis of TBG, T4 uptake, and secondary release of T4 and T3 into the circulation. Abnormalities in serum thyroid hormones are frequently found in cases of cirrhosis, acute hepatitis, and chronic liver disease [21, 70, 71].

In cases of cirrhosis, the most common finding is low TT3 and FT3 concomitant to elevated rT3. The serum relation TT3/rT3 is inversely associated with the severity of the disease [72]. Free T4 may be increased, while TT4 can be decreased due to low TBG and albumin synthesis. TSH is usually normal or mildly increased, but the patients have a euthyroid clinical presentation [21].

The alterations found in acute hepatitis are different from other forms of liver disease. Elevated TBG is a consequence of its hepatic release as an acute phase protein. Consequently, total T3 and T4 are usually elevated, while the free form of thyroid hormones remains within normal range. A mild elevation of rT3 can be found, while TSH is most often normal [19].

In chronic liver diseases thyroid hormone imbalances resemble more those of acute hepatitis than the ones found in liver cirrhosis. Examples of studied liver diseases are primary biliary cirrhosis and autoimmune hepatitis. In these, serum TBG levels are high, as are TT4 and TT3. However, serum FT3 and FT4 are low [73]. Difficulties in hormone assessments occur due to the fact that both conditions have an autoimmune basis and exclusion of autoimmune thyroiditis is warranted [7]. Noteworthily, thyroid hormone abnormalities found in these diseases are not associated with prognosis [37].

### 4.6. Respiratory Diseases

Some authors have found evidence of NTIS in chronic obstructive pulmonary disease. Karadag et al. [4], in a study involving 83 patients in stable clinical condition, 20 with acute exacerbations, and 30 healthy individuals, observed that patients with stable disease had FT3 levels 25% lower than healthy volunteers, without differences in TSH or FT4. The fall in FT3 levels was associated with increases in interleukin 6 and tumour necrosis factor alpha. Acute exacerbations lead to further decreases in FT3 levels and a small decrease in TSH levels, all of which returned to basal levels after clinical stabilisation.

During tuberculosis infection, one study showed that T3 levels are low in more than 50% of the patients, with no change in TSH, T4, or serum TBG levels. After a short period of treatment, T3 levels were restored to normality and TBG levels rose to supernormal levels when compared to a control group taking prophylactic treatment [74]. Although this could have been attributed to drug induced hepatitis, only one patient was diagnosed with the condition.

### 4.7. Diabetes Mellitus

Alterations of thyroid hormone axis have been demonstrated in patients with diabetes mellitus (DM). Some authors found decreased serum TT3 and, in a few cases, TT4, concomitant to increased rT3 and low or inappropriately normal TSH [75]. Comparable abnormalities have been found in patients with type 1 DM, particularly in the presence of poor glycaemic control, as reflected by higher glycated haemoglobin levels [76–78]. Similar correlations were found in patients with type 2 DM, especially when the glycated haemoglobin was above 12% [75].

An interesting study conducted by Kabadi [3] in patients with recently diagnosed type 2 DM and glycated haemoglobin above 10.8% found elevated rT3 and low T3 levels, but these abnormalities were fully reversed upon restoration of good metabolic control.

As both type 2 DM and NTIS present a strong inflammatory pathogenesis, it is not surprising that subclinical inflammation present in obesity and type 2 DM is correlated with serum thyroid hormone levels. A recent work has shown that rT3, waist circumference, and high-sensitivity C-reactive protein were interrelated in patients with type 2 DM [17]. In another study, a subset of patients with type 2 DM, serum rT3 was elevated only in those with previous cardiovascular disease, such as angina or stroke. These were also the patients showing the greatest increase in hs-CRP levels [30]. In both studies, no relation between HbA1c and thyroid hormones was found. Therefore, poor glycaemic control might not be solely responsible for thyroid hormone abnormalities in patients with DM. In fact, a recent study found that abnormalities in FT4/rT3 and FT3/rT3 ratios in patients with type 1 and type 2 diabetes were linked to higher serum concentrations of proinflammatory markers associated with NTIS, such as IL-6 [79], while HbA1c was related to higher FT4/FT3 only in patients with type 1 diabetes. The data suggests that in diabetes mellitus the main pathophysiological process may be related to abnormal deiodinase activity. Abnormalities in type 2 deiodinase have been related to a higher incidence of type 2 diabetes [80] and increased insulin resistance [81].

### 4.8. Psychiatric Illness

Abnormalities in thyroid hormone profiles are not uncommon in patients with psychiatric illnesses, especially if hospitalization is required. The main disorders associated with NTIS in these patients are posttraumatic stress disorder, schizophrenia, and major depression [82–84]. Psychiatric disorders are unique in that they present high T3 and/or TSH levels, as opposed to the low thyroid hormone and TSH levels found in other acute and chronic diseases.

In posttraumatic stress disorder, patients may present mild increases in serum total T3 levels but FT3, FT4, and TSH are usually normal [82]. In those admitted due to severe psychosis, about 1 in 10 will present thyroid function abnormalities [83]. The most common is high T4 and TSH, simulating the profile of patients with TSH-producing pituitary tumours or resistance to thyroid hormone. Opposite to what happens in the latter two conditions, thyroid hormones and TSH usually normalize spontaneously in 7 to 10 days in acute psychosis and a conservative approach is recommended when evaluating such patients [85].

Patients with major depression may have TSH and T4 concentrations within the normal range, although showing higher levels when compared to matched controls, as well as low TRH-stimulated TSH levels [84]. These may be a result of diminished TRH mRNA expression in the hypothalamus.

## 5. Treatment

Treatment of thyroid hormone abnormalities in patients with NTIS is as controversial as its physiological interpretation. Few clinical studies are available to assess thyroid hormone replacement in this situation and almost all were conducted in critically ill patients.

One study assessed the effects of replacement with 150 mcg/day of thyroxine in four doses divided in 2 days in patients with acute renal failure. The only difference encountered was in TSH levels and the treated group showed higher mortality [86].

Of particular interest are the studies conducted in patients with heart diseases subjected to coronary revascularization, which showed increases in cardiac output and lesser need for vasopressors during recovery but no other effects [87]. Patients with advanced heart failure responded to T3 administration with decreases in serum norepinephrine, aldosterone, and atrial natriuretic peptide as well as decreased heart rate and improved left ventricular function without major side effects [88]. It is noteworthy that treating systemic inflammation can also prevent the abnormalities typical of NTIS, as was demonstrated in a recent study in patients with acute myocardial ischemia [63].

Thyroid hormone replacement in NTIS prevents the TSH elevation that is expected in the recovery phase of the original disease [89]. Since decreased conversion of T4 to T3 is present in most cases of NTIS, some authors have advocated that if treatment is warranted, it should include T3 or a combination of T4 and T3 [90].

It is possible that treatment in acute situations where decreased T3 is believed to be a proper adaptative response to stress may be harmful, while thyroid hormone replacement in conditions of chronic low T3 may be beneficial, especially in patients with heart diseases. However, it is noteworthy that there are no randomized controlled clinical trials assessing the effects of thyroid hormone supplementation in such situations and treatment of these patients is therefore not recommended.

## 6. Conclusion and Future Perspectives

Thyroid hormone abnormalities characterizing NTIS in different clinical setting are complex and have a multifactorial origin. There is considerable variation in laboratorial presentation, depending on the original disease. As is observed in patients with acute and more severe diseases, the intensity of thyroid hormone imbalances in patients with chronic diseases represents the severity of the underlying disease and keeps an intimate correlation with the prognosis in most cases. Thyroid hormone replacement to such patients is still largely debatable, as most studies were conducted in patients with acute exacerbations. Patients with heart diseases are most likely to benefit from such treatment, but this should be confirmed in appropriately powered clinical trials. Treatments targeting other aspects of NTIS such as systemic inflammation may show benefit in preventing the occurrence of thyroid hormone abnormalities and also warrant further research.

## 7. Clinical Case

A male patient, 61 years old, treated for congestive heart failure since 2008 due to a myocardial infarction, had for the last 6 months experienced progressive worsening of dyspnea and lower limb oedema, despite frequent optimization of his medication. Laboratorial investigation for his worsening symptoms revealed a TSH of 4.3 IU/L (RV: 0.5–4.5 IU/L), free T4 21 pmol/L (RV 10–23 pmol/L), and free T3 2.5 pmol/L (RV 3.5–6.5 pmol/L). Echocardiography showed a dilated heart, a left ventricle ejection fraction of 28%, and moderate pulmonary hypertension. He was a smoker for 30 years and had quit 10 years before. Other relevant comorbidities included hypertension and hypercholesterolemia. His lipid panel and ambulatory blood pressure profile were within targets. His clinician referred him for evaluation of a possible hypothyroidism that could be contributing for the deterioration of cardiac function as well as evaluation for treatment.

Initial evaluation yielded negative antithyroid antibodies and a magnetic resonance image of his pituitary revealed no abnormalities. The low free T3 concomitant with normal FT4 and TSH was interpreted as a form of NTIS in this patient and as a marker of poor prognosis given the history of heart failure and rapid progressing symptoms in the last months. Treatment with T3 was considered, but as there is no conclusive evidence that treatment with thyroid hormones could improve the condition or even survival, it was decided for observation and recommended for further investigation into the cause of cardiac decompensation.

Coronary angiography revealed no new obstructions and the patient had no signs or laboratorial evidence of infections. Eventually, a computed tomography revealed a pulmonary embolism as the cause for his worsening symptoms. The patient was admitted for initiation of anticoagulant treatment and showed progressive clinical improvement until discharge 7 days later. At the end of anticoagulant treatment, his dyspnea was back to previous levels and the echocardiography-estimated right ventricle systolic pressure had improved. A new thyroid function test was ordered and showed TSH 4.1 IU/L; FT4 17 pmol/L; and FT3 3.1 pmol/L. Despite the increase in serum FT3 after treatment of pulmonary embolism, its levels remained below normal values, probably due to the long term, irreversible heart failure.

## Figures and Tables

**Table 1:** Summary of thyroid hormone abnormalities found in noncritically ill patients.

	Total T3	Free T3	Reverse T3	Total T4	Free T4	TSH
Caloric deprivation	↓	↓	↑	↓	*⇔*	*⇔* or ↓
Heart failure	↓	↓	*⇔* or ↑	*⇔* or ↓	*⇔*	*⇔* or ↓
HIV infection	*⇔*	*⇔* or ↓	*⇔* or ↓	*⇔*	*⇔* or ↓	*⇔*
Renal diseases	↓	*⇔*	*⇔*	*⇔* or ↓	*⇔* or ↑	*⇔*
Liver diseases	*⇔* or ↑	↓	*⇔* or ↑	↑	*⇔* or ↓	*⇔*
Pulmonary diseases	*⇔*	↓	*⇔* or ↑	*⇔*	*⇔*	*⇔*
Diabetes mellitus	↓	↓	*⇔* or ↑	↓	*⇔* or ↑	*⇔*
Psychiatric illnesses	↑	*⇔* or ↑	*⇔*	↑	*⇔* or ↑	↑

*⇔*: normal.

↑: increased.

↓: decreased.
